# The Nature of Trauma Informed Care in the Treatment of Men Convicted of a Sexual Offence With Trauma History

**DOI:** 10.5964/sotrap.13991

**Published:** 2025-09-19

**Authors:** Sarah Senker, Anne Eason, Kieran McCartan

**Affiliations:** 1 School of Social Sciences, University of the West of England, Bristol, United Kingdom; Centre for Criminology (Kriminologische Zentralstelle – KrimZ), Wiesbaden, Germany

**Keywords:** trauma informed practice, trauma informed care, people with sexual convictions, risk management, dual status, victimisation

## Abstract

Individuals who have committed sexual offences are more likely to have experienced trauma or childhood adversity than the general population (Kahn et al., 2021). Further, individuals who were convicted of a sexual offence perceive a strong connection between their own victimisation and their sexual offending (Grady et al., 2022). This initial scoping work, funded by the University of the West of England in the United Kingdom, involved in-depth qualitative interviews with 20 professionals and a roundtable including academics, policy makers and front-line practitioners from His Majesty’s Prison and Probation Service, NHS and third sector organisations. All participants had a history of working with individuals with a sexual conviction and a history of trauma or had been involved in academia in this area. The work identified an inconsistent use of the term ‘trauma informed’ across the sector and a gap in trauma informed care (TIC) for individuals with a ‘dual status’ as both a perpetrator of sexual abuse and victim of trauma. This was especially prominent for adult men relative to females or young people with sexual convictions. Individuals with a ‘dual status’ are particularly disadvantaged in having their trauma acknowledged and addressed if they are male. The extent that true TIC can be implemented across the justice system is challenged by the necessities of managing risk. Further research is required to better understand the experiences of people with lived experience and to explore the impact of TIC on reintegration and reoffending.

## Highlights

- Trauma histories are present in men convicted of a sexual offence.
- Having been caught and convicted of a sexual offence often means that the individual’s victim status is compromised.
- More work needs to be done to develop trauma informed approaches, within the criminal justice system, for men convicted of a sexual offence.

Understanding what contributes to a person’s offending behaviour enables criminal justice practitioners to prevent or reduce offending, respond effectively, and manage people at risk of committing a future offence (McCartan, 2020). One aetiological factor gaining increasing recognition and prominence is the experience of trauma in the lives of people with a criminal offence, and the role it may play in the genesis and maintenance of offending behaviour.

There is a high prevalence of trauma and childhood adversity in people who have committed sexual offences, relative to the general population (Kahn et al., 2021). Importantly, research has shown that there is also a link between individual’s own victimisation and their sexual offending (Grady et al., 2022). Previous work has indicated the impact of addressing trauma in those with sexual convictions (Perry et al., 2018; Wright et al., 2024). For example, perpetrators who had eye movement desensitisation and reprocessing (EMDR) therapy reported a reduction in frequency and strength of sexual arousal towards children and this was maintained three years later (Wright & Warner, 2020). However, whilst there is increasing theoretical support for care and interventions that acknowledge the trauma in the lives of people with an offence history, there is a relative paucity of current research which affords consideration to the extent this is being undertaken with people who have sexual convictions, the way terminology is used and understood across the justice and health sector, and the impact it has on reducing risk of harm. This is reflected in the insufficient and inconsistent provision of trauma informed interventions for high risk of harm individuals who have committed a sexual offence and thus their ability to achieve desistance.

A critical review by Bargeman, Smith, and Wekerle (2021) found that a common theme across several sectors (child welfare, education, juvenile justice, health and multiple sectors) was a lack of understanding regarding what trauma informed care is and how to implement it into services. This suggests more work is needed to raise awareness and assist implementation into services. The term ‘trauma informed’ care or practice has become increasingly popular over recent years in a variety of services including those in the criminal justice sector (Becker-Blease, 2017). Traced back to the work of Harris and Fallot (2001), the term is distinct from ‘trauma therapy’, ‘trauma awareness’ and ‘trauma responsive’ and encompasses a wide range of initiatives that are considerate of the sensitivities around trust, power and stigmatisation. Harris and Fallot (2001) outline five key principles underlying trauma informed care: safety, trustworthiness, choice, collaboration and empowerment and are clear that this pertains to those receiving and providing services. The model considers individual agency and brokering the barriers to empowerment by drawing on innate skills such as self-insight and facilitating informed choice. To do this, trauma informed care often requires an organisational shift to be truly embedded in work practices, procedures, protocols and physical environments and only with such a holistic approach can it prove effective to those engaging in it, at a practitioner and service-user level.

Therapy to address trauma can include Eye Movement Desensitisation and Reprocessing (EMDR) or Trauma-Focused Cognitive Behavioural Therapy (CBT) amongst others and this is often used interchangeably when discussing trauma informed care despite being distinct modes of therapeutic intervention. Becker-Blease (2017) warns of adopting a ‘one size fits all’ approach emphasising how an initiative may have a positive impact on one person but may adversely, even re-traumatise another. Thus the interchangeability of different therapeutic approaches can be much more effective than referring to whole systems, environments or singular approaches.

The current study sought to explore, from the perspectives of professionals working across health and justice, what currently exists in the United Kingdom, by way of trauma informed care (TIC) for those individuals who have a conviction for a sexual offence but have also experienced trauma themselves (pre-dating their current offence). For the purposes of this study, trauma was defined as any form of abuse or adverse life experience (excluding medical trauma e.g. brain injury) that occurred before the onset of sexual deviance or sexual offending. The person could have been any age when this trauma occurred (not solely adverse childhood experiences). The term TIC was defined as ‘the creation of an environment where a person who has experienced trauma feels safe and can develop trust’ (Fenney, 2019). The key research questions were to identify what exists for this group, how the term trauma informed is currently used amongst professionals and the extent this is consistent as well as the perceived importance and challenges associated with trauma informed care for this cohort.

## Method

The method adopted for this study was qualitative. Tewksbury (2013) notes the suitability and superiority of qualitative approaches within criminology because of its ability to generate rich, enhanced understandings of complex topics. Recruitment for in-depth interviews was via snowball sampling. The research team identified key stakeholders within the sector, individuals and organisations working with individuals with sexual convictions. This included statutory organisations as well as third sector providers. Key stakeholders were contacted and sent an information sheet about the study and gave informed consent to take part in an in-depth interview. At the end of the interview they were also asked if they could identify any other key professionals to invite to engage. Interviews were audio recorded and transcribed verbatim, removing all identifiable information. Aggregate qualitative interview transcripts were analysed by the primary researcher as well as other members of the research team for inter-rater reliability. The method of analysis was thematic adhering to Braun and Clarke’s (2006) inductive six step approach ensuring an inductive rather than deductive approach. The initial themes that were coded and derived were presented to contributing stakeholders at a Roundtable event as a way of sense-checking findings and inviting further discussion. The data from all transcripts and the roundtable event were then analysed for a final time, thematically, forming the findings presented in the current paper.

We considered the quality of the data using Yardley’s (2000) criteria of commitment and rigour; transparency and coherence; impact and importance.

## Findings

The findings presented here relate to three key, overarching themes that emerged from the data; inconsistent use of terminology; the pains of ‘dual status’ and the foundations of good practice.

### Theme 1: Inconsistent Use of Terminology

Interviewees acknowledged and reflected that ‘trauma informed’ has become a ‘buzz word’ in recent years, noting a proliferation in its use alongside a lack of clarity and consistency over what it ‘actually means’. At the heart of the issue, interviewees reflected on how the term ‘trauma’ is contentious, raising debates about whose trauma it is to define, at what point a life experience becomes traumatic and how this can vary dependent on an individual’s resilience, protective factors and personal interpretation of an event (or series of events).

*‘You're probably spending a lot of your time defining what trauma means...they find themselves in a situation whereby they feel out of control and in danger. The trauma is that they are out of control of the situation they're in, and they are fearful. That's what I think in very, very simple language, to my understanding, is what trauma is’* (Interview Participant)

Interviewees discussed a sense of powerlessness as a key factor in experiences being internalised as traumatic. This is important in the context of sexual offending, but also in trauma informed care which has empowerment as a central pillar.

*‘I wouldn't call it trauma, I would call it powerlessness. Now, the thing about powerlessness is that it can come in all sorts of different forms...feeling that you are significantly disempowered to a point which has become toxic. Now that, in my view, is at the root of most sexual offending’* (Interview Participant)

Interviewees working on the front-line to deliver interventions to people convicted of sexual offences, reflected that professionals will often define individuals lived experience as a trauma, but this does not necessarily mean that the individual experienced it as traumatic, nor that it affects their functioning. Several interviewees discussed examples and cases where, especially in sexual abuse and sexual offending, the individual had experienced sexual abuse as a child but internalised this as a pleasurable and arousing experience which later contributed to their offending behaviour.

*One of the issues we have is that people will often not label what's happened to them in their childhood as traumatic. They'll normalise their family set up and what experiences they've had, but they share things that we would think may be traumatic or may have been traumatic’* (Interview Participant)

*‘You see a lot of people who have coped with their own abuse by sort of suppressing the negative aspects of it, that were too difficult to deal with, and just focusing on the positive ones. So maybe the grooming behaviour, where they felt quite close or quite special or connected to the abuse…so touching that felt quite pleasurable, that wasn't too invasive or painful, they block out the negative bits and focus on those bits. And remember that they enjoyed it, and they were sexually aroused at the time’* (Interview Participant)

This therefore posed discussions around whether services should use the word trauma with the individuals they work with, recognising it as a value laden term.

Discrepancies around the use of the term trauma, clearly have a resultant impact on how term trauma informed practice is shaped. Relatively few interviewees were cited on the origins and tenets of trauma informed care, nor the work of organisations who offer accreditations for being ‘trauma informed’. Some interviewees acknowledged a distinction between a trauma informed environment, a trauma informed service and a trauma intervention/therapy but others referred to these concepts interchangeably.

*‘I'm going to be honest, I think there is a misconception about what a trauma informed approach is, and so what happens is because there is misinformation and a misunderstanding, people think that trauma informed is an intervention, as opposed to a framework and culture change and systems change’* (Interview Participant)

Further, the challenges noted around terminology and definition were said to contribute to a lack of prevalence data within services. Interviewees stated that anecdotally they know a high number of individuals with sexual convictions have trauma in their background but they do not routinely collate data on this, in part because of the subjectivity and sensitivity around it. However, where organisations had undertaken their own analysis on people they support they reported high correlations.

*‘When we've looked at the people who've disclosed to us that they've been sexually abused out of men who have sexually offended against children, that we found that if they had offended against children who were 13 or younger, sometimes 70% of them disclose being sexually abused themselves as a child. And that when you went to people who had offended with people over 13, that the rates went down’* (Interview Participant)

### Theme 2: The Pains of ‘Dual Status’

Interviewees were asked what currently existed for individuals with convictions for sexual offences, that had also experienced trauma. The findings indicated that, for adult men especially, they are a somewhat forgotten demographic with an emphasis on reducing risk and addressing offending behaviour, perhaps to the detriment of acknowledging or addressing trauma and the role this could have on prevention. In the community, interviewees discussed that services for men who have a history of trauma and sexual convictions are not readily nor consistently available. The findings allowed a greater understanding of the trauma informed support available to individuals entering the justice system. Ultimately the findings reinforced that the UK criminal justice system is focused on protecting the public and managing risk which can make it difficult to accommodate trauma informed approaches. One interviewee highlighted the focus in prisons on ‘containment’ and ‘punishment’ relative to secure hospital settings. Another interviewee reflected on the challenges of acknowledging individual trauma in offender behaviour groups programmes.

*‘If they disclose sexual abuse, you acknowledge the difficulty that would have been... but essentially, you're shutting them down. It doesn't obviously say that in the manual, but that's what it very much feels like as a practitioner, that you're saying well, thanks for sharing that but we're actually interested in what you've done to somebody else instead, and it just feels really unethical. And, I think there's sort of a preciousness about exploring their experiences. And there's very much an emphasis on holding them to account for what they've done. But there's not that courtesy around what's happened to them...but that's disregarded in lots of ways.’* (Interview Participant)

However, some interviewees indicated a more recent shift to rehabilitation and compassion with the introduction of initiatives in His Majesty’s Probation and Prison Service such as ‘the five minute intervention’, ’key worker scheme’ and a focus on relational aspects such ‘as every contact matters’. It was acknowledged that a very small number of individuals in this cohort would attend a therapeutic wing or community, or access EMDR therapy. However, the percentage of people that this will be offered to was hypothesised to be fractional compared to need. Therefore, interviewees discussed that although EMDR or therapeutic communities may be the gold standard they are currently not routinely able to be accessed by people with ‘dual status’.

*‘They do not exist these treatment pathways, or if they do they're so rare, and so obscure that they might as well not exist. If something's only gonna get hit one in 100 times, I'd almost say I'll tell you what, forget it. Because what about the 99 people that don't get it?’* (Interview Participant)

Further, interviewees were able to reflect on how prison can be both trauma inducing and trauma reinforcing in itself for both staff and prisoners, impacting the delivery of trauma informed care.

*‘It's really difficult for those organisations to do it authentically, because they're ultimately punitive. The staff are overworked and so if you can't be trauma informed towards your staff, if you're traumatising your staff, then it's difficult for your staff to be trauma informed’* (Interview Participant)

Interviewees also acknowledged that for people in this ‘dual status’ cohort, the restrictions imposed on them (such as disclosing new relationships or where they could reside) also limited people’s ability to address their trauma and successfully reintegrate into society.

*‘The UK is one of the countries with the highest number of restrictions we impose on people with convictions for sexual offences. We monitor them to death, and from the day they come out of prison... the surveillance...being on the register, all of those conditions. When you compare us to the Scandinavian countries they don't have half of the restrictions we have yet they don't have more sexual crime than we have okay. So it's an interesting thought. I've been very, very interested in this quaternary prevention stuff and linking that to trauma. I just think we are constantly just replaying the trauma to this person by all these restrictions? How are they treated? When you have to report at the police station to make an application to leave the country or change your address or all that kind of stuff. How is that person treated there by the police? We just reinforced that trauma wheel the whole time with all of that’* (Interview Participant)

The role of the wider public narrative or view on sexual offences was also noted by interviewees as an important and compounding factor. An overarching public resistance, or worse, distaste to seeing people with sexual convictions as people who may have experienced trauma was reported to act as a challenge or barrier for services and systems to acknowledge trauma in this group.

*‘Very often perpetrators are not seen as suffering trauma or having had traumatic upbringings or adverse childhood experiences etc. so we don't want to see them as survivors, we want to see them as perpetrators, that is society and society wants to be as punitive as they possibly can. It's the group of offenders that is treated in the most punitive way of anyone. We're prepared to forgive robbers and murderers. But just don't abuse a child or be a rapist;* (Interview Participant)

Overall, interviewees described a prevalent scenario where people with sexual convictions rescind their right to be considered a victim when they offend – especially if it’s in a similar way to the way they were victimised. Further, interviewees observed differences in how men, women and young people within ‘dual status’ cohorts are regarded and treated. It was proposed, by interviewees, that adult males are less likely to a) feel comfortable about discussing trauma and b) have access to trauma informed support that recognises their status as a victim and perpetrator. Therefore, interviewees concluded that that there are differences in how trauma is categorised and responded to by professionals, based on gender of the perpetrator.

*‘I definitely believe the gender stuff is a big issue for me around trauma. I think there's such a strong anti-male feeling at the moment. It's like men are not allowed to have had a trauma’* (Interview Participant)

### Theme 3: The Foundations of Good Practice

In light of the discussions above, acknowledging a lack of consistency over the term trauma and trauma informed care, and a lack of consistent or specific support for those with ‘dual status’, interviewees considered what ‘good’ should look like. Firstly, interviewees acknowledged that the wider context and broader circumstances of giving trauma informed care or trauma therapy are important. Trauma informed care is more than an individual interaction or exchange, more than an acknowledgement of the importance of trauma. Rather, the environment, the strategic leadership of an organisation was reportedly required to buy in to trauma informed principles, to invest in its implementation over a long time period so it is ‘*almost seeping out the walls and every fabric of every policy and decision’.*

*‘Working with individuals doesn't mean sort of seeing them for counselling once every three days or being in a group or a therapy group. It means working with them when they're having their dinner, when they're having their lunch, when they're playing table tennis, being interactive in a humanistic way and providing a setting within which that person, probably for the first time, begins to feel that they're relatively respected, relatively affirmed, relatively recognised. And it doesn't have to just be in the one-to-one counselling or the group therapy or whatever. It happens in the way that they're treated all the time’* (Interview Participant)

One interviewee articulated that the ‘intervention’ is the last thing in the chain that is implemented requiring a robust, thorough infrastructure and governance system sitting behind it. The time taken to create a trauma informed culture is lengthy which can act as a barrier for investment and present challenges in terms of evidencing outcomes.

*‘There has to be a real process to it and the intervention is almost the last thing that comes on board. So you need to do everything else first, which is get buy in from the management. If it's prison, like your number one governor and all the senior management team have to be brought in and they have to know what it's about. So doing training on the principles of trauma informed practice and what they actually mean they're not just words that you put down in your bullet points in your business plan but what it means to act, practice in that way, within an organisation and criminal justice organisation’* (Interview Participant)

*‘There's a danger of things becoming sort of academic terms that sound good but how they translate into practice... I think the operationalization of that is patchy’* (Interview Participant)

In acknowledging the challenges in implementing true trauma informed care, interviewees simultaneously discussed the ‘danger’ of dealing with trauma in a broader environment that does not support this, leaving individuals ‘uncontained’ or ‘destabilised’.

*‘Part of delivering EMDR means that we have to have a structure where patients can access and engage with that treatment, because EMDR is a really good intervention, but it's also a highly destabilising intervention. And so we need to make sure that if we're going to do that the prison needs to support us with certain things. And some of it is around how are we going to manage this patient? Are we going to provide them with a single cell? Are they going to move wings? Are we going to make sure that they're not going to be transferred halfway through the treatment? Are they ready?* (Interview Participant)

Concerns were also expressed by interviewees that organisations and environments that try to achieve ‘quick wins’ may also do further harm to individuals.

*‘If somebody's been traumatised, the feeling they have being the victim of that trauma is complex, it's painful, it's angry making, it's shaming, all of those core difficult emotions. If you've been traumatised. then you're not going to work with them in six sessions or 12 sessions. With all due respect, it's nonsense’* (Interview Participant)

Interviewees were clear that trauma informed care requires support and comprehensive understanding from the top down including senior management and leadership teams. However, they also were keen to discuss the role of Government support alongside wider societal and community buy in.

*‘The victim and perpetrator are in the same person. And that's, once staff begin to realise that I think their attitude changes, because they won't just think that this person is an evil bastard, they'll think he's an evil bastard, who actually was treated pretty badly when he was a child. You know, their whole attitude changes. So that is the shift that's very difficult for some people to make. And if you don't have the prison governor and some senior people supporting that shift and attitude, on the ground, the face-to-face staff and prison officers and the nurses and stuff will find it very, very difficult to sustain that view. But if the governor and the senior staff take that view, they can support the supervision group or the consultation or the reflective practice group, whatever to help them begin to think like that’* (Interview Participant)

Compassion emerged as a theme throughout discussions, for staff as well as people with ‘dual status’, acknowledging that trauma informed work does not occur within a social vacuum, but rather is an exchange and therefore all involved within that exchange require support. Therefore, best practice when implementing trauma informed care extends to ensuring embedded policies around staff well-being, considering how they cope and ensuring they are supported. Interviewees discussed the challenges associated with staff burnout and churn in criminal justice settings. They reflected that this has an exacerbated impact if staff, who have been trained in trauma informed approaches, move on.

*‘What I have found in the years that I've been working at HMP X is that we also have a high level of highly traumatised staff working in those environments. And a lot of the work that I was trying to do through the MDT was really to bring forward staff's responses, and staff's experiences to what they were exposed to. So when we have, you know, staff responding to women that are self-harming and I'm talking about the severe self-harm on a day to day basis, it's going to have an impact. But the culture in the prison doesn't allow staff to make themselves look vulnerable. And that's a big problem’* (Interview Participant)

Importantly interviews also discussed the impact of dealing with trauma on overall well-being of people with sexual convictions but also how they make sense of their offending.

*‘We did EMDR to reduce the trauma symptoms, it wasn't with the intention of reducing his risk, it was because he needed this for that reason. But what I found was that he was saying my sexual feelings have changed and actually, I want to think back to my abuse, now it feels very different, I'm not sexually aroused by it anymore…so at the end of EMDR, he no longer felt sexually aroused when he thought about his own abuse. He no longer felt sexually aroused when he saw a child in the street, as well as all the other PTSD symptoms, having been limited to no more flashbacks or intrusive thoughts or nightmares. You know, he said he had the first good night's sleep without any nightmares since he was 13. At this point, he's about 27 years of nightmares every single night’* (Interview Participant)

## Discussion

Based on the findings of qualitative in-depth interviews with professional stakeholders, across a range of settings, we have explored the fact that trauma informed care is not consistently used nor well understood. We have seen how terms such as ‘trauma therapy’ or ‘trauma informed’ are used interchangeably as well as the difficulty in creating whole systems change for best practice. The interviews outlined that for adult men, with a history of trauma and sexual convictions, their trauma is not routinely addressed nor acknowledged despite it being prevalent and having positive implications for offending if resolved.

For those people with sexual convictions who have untreated historical trauma, their dual identity can manifest in a cyclical pattern of heightened risk presenting with a range of challenges, not least the inability to achieve long-term desistance. Kemshall (2021, p. 9) suggests that ‘desistance and risk management can be understood as two sides of the same coin’ that when working consecutively can attain public protection through non-offending. So, whilst there may currently be limited research into the benefits of providing trauma informed approaches to people convicted of a sexual offence, the impact of the subsequent lack in interventions, is extensive. Longer-term suffering for those who are victims of trauma and have harmed others require greater resource requirements to manage their behaviour safely (e.g. resource follows risk, HMPPS, 2022). Further a higher probability of potential reoffending is costly financially, psychologically and in terms of public confidence.

### The Juxtaposition of Trauma Informed Care and People Who Have Committed a Sexual Offence

The findings from the qualitative research show a recognition and a willingness to work with people convicted of a sexual offence in a trauma informed way, but currently, on a systematic level, the practice doesn’t reflect this individual desire or intention. Trauma informed working is a relatively new concept in criminal justice and, the current findings suggest that organisational thinking has not yet matured to reflect the additional nuances of implementation that are required in these settings relative to health and public health (Senker et al., 2023). This is especially the case with people convicted of a sexual offence. As acknowledged in the literature and current interviews, the high prevalence of trauma in this cohort can pose a professional and political challenge to policing, prison, and probation with a careful balance of compassion, rehabilitation and punishment. The findings show a willingness in a desire to change prison culture which is in line with research around rehabilitative culture in prisons (Durr, 2020; Mann et al., 2019; Trauma informed prisons project, Royal Society for Public Health, 2023) and HMPPS policies indicating that prison needs to be trauma informed (HMI Probation, 2022). However changing prison culture is not easy nor swift. In secure settings being trauma informed requires a place based, cultural shift in attitudes and behaviours. Further, whilst a systemic approach is required consideration must also be given to each individual prison population, staffing and local community. Further, the desire and willingness to work in a trauma informed, empathetic and considered way with people convicted of a sexual offence is slowed down, and potentially derailed, by socio-political factors and overarching public narratives. This highlights the need to produce an evidence base on which to make informed decisions and develop good practice which is robust in the face of challenge.

### Trauma Informed Working Reinforces the Bio-Psyche-Social Model and the EpiCrim Framework

Ultimately, the research reinforces the emergence of a trauma informed approach to working with people convicted of a sexual offence throughout their criminal justice system journey, reinforcing the bio-psycho-social model used by HMPPS and the EpiCrim Framework (Kemshall & McCartan, 2022; McCartan, 2020, 2022). Being trauma informed means taking a person centred, empathetic approach focusing on behaviours and not offences, that is future facing. Additionally, being trauma informed reinforces the therapeutic principles of the programs used in HMPPS and the strengths-based thinking that underpins them. Being trauma informed works across the four stages of the socio-ecological model (individual, interpersonal, community, and societal) and as well as being rooted in the relapse prevention practice (tertiary and quaternary prevention). It also has implications for preventing first time offending (primary and secondary) as it indicates that if people convicted of a sexual offence have trauma in their lives pre offending it advocates for better early intervention, better victims services, and more attention needing to be focused on life course development and attachment. Being trauma informed, as indicated by the professionals in this paper, is important because it supports the reduction of offending and promotes social inclusion and integration.

### Conclusion

This work explored the understanding and use of the term trauma informed care in the UK justice system, with particular consideration for those individuals who have committed sexual offences and have experienced trauma pre-dating this offence. Interviews with 20 stakeholders working across health, justice and academia indicated inconsistencies in the use and application of this term and a significant disadvantage for adult men who have a history of trauma and sexual convictions in addressing this. The paper argues that whilst trauma informed care is a long-term, organisation wide approach, the correlation between trauma histories and sexual offending suggests there is merit in implementing this. The discussion makes links between risk management and the role addressing trauma can play in reducing risk and promoting desistence. The findings present a foundation to argue for a movement which ensures consistency in the use of the term trauma informed, allowing greater access to trauma work and spaces which afford consideration to the role of trauma within the justice system. This would need to be embedded in a wider compassionate culture which also sees staff supported in a trauma informed way.

## Data Availability

The data that support the findings of this study are available from the corresponding author, upon reasonable request.
